# Comparison of the colchicine concentration between different matrix; plasma, leucocytes, ficoll solution measured by ESI LC-MS/MS

**DOI:** 10.1186/1546-0096-13-S1-P137

**Published:** 2015-09-28

**Authors:** H Gul, E Demirkaya, B Eser, T Honca, FN Felek, D Simsek

**Affiliations:** 1 Gulhane School of Medicine, Ankara, Turkey

## Introduction

Colchicine is the main therapeutic drug which is used for Familial Mediterranean Fever (FMF) and acute gout attacks. In a limited study, the leucocyte colchicine level has been implicated to be important for monitoring of drug level. It will be important especially in the drug resistant patients. Therefore, we aimed to investigate the usability of different matrix for the monitoring of colchicine level.

## Methods

We used Agilent model 6420 LC-MS/MS using electrospray ionization (ESI) to measure colchicine level. We used whole blood as a specimen, first we obtained plasma by centrifugation, second we collected leucocytes using Ficoll-Hypaque, and the last we separated Ficoll during leucocyte isolation. We added colchicine into whole blood at a concentration that to obtain 10 ng/ml final concentration. Tegafur was used as an internal standard. We lysed leucocytes by TritonX (1/1000: v/v). Colchicine was extracted from the all three matrix with 3 fold volume of the matrix of a dichloromethane/isopropanol/ethyl acetate mixture (1:1:3;v/v). After phase separation by centrifugation, the organic layer was transferred and evaporated to dryness. The residue was reconstituted with mobile phase and injected into LC-MS/MS. LC flow rate of 0.5 ml, the phase gradient of 10 mM ammonium acetate + 0.1% formic acid+water for phase A and the mixture consisted of 0.1% formic acid in methanol for phase B were used.

## Results

We found that colchicine levels of the three matrix were correlated each other. However, the necessary volume of plasma samples, 200 microlitre, was lower than Ficoll-Hypaque solution (1 mL) which was used for leucocyte separation. Plasma specimens seem more clear and suitable than serum specimens. Colchicine concentration of leucocyte obtained from 1 mL of whole blood is nearly half of plasma level. In addition, tegafur, IS, was correlated with colchicine level well in all matrix.

## Conclusion

We showed that plasma, Ficoll-Hypaque, and leucocyte could be used for the monitoring of colchicine level in patients. However, we do not know which matrix is the best suited for the monitoring of colchicine level in resistant patients. Therefore, further studies are needed to clarify this point in a large patient population comprising resistant patients as well.

